# PG545, a dual heparanase and angiogenesis inhibitor, induces potent anti-tumour and anti-metastatic efficacy in preclinical models

**DOI:** 10.1038/bjc.2011.11

**Published:** 2011-02-01

**Authors:** K Dredge, E Hammond, P Handley, T J Gonda, M T Smith, C Vincent, R Brandt, V Ferro, I Bytheway

**Affiliations:** 1 Progen Pharmaceuticals Ltd, 16 Benson Street, Toowong, Brisbane, QLD 4066, Australia; 2 Molecular Oncogenesis Group, The University of Queensland, Diamantina Institute, Princess Alexandra Hospital, Ipswich Road, Buranda, QLD 4102, Australia; 3 Centre for Integrated Preclinical Drug Development, The University of Queensland, St Lucia Campus, Brisbane, QLD 4072, Australia; 4 vivoPharm Pty Ltd, 1-5 Wakefield Street, Kent Town, SA 5067, Australia

**Keywords:** heparan sulfate, angiogenesis, heparanase, anti-tumour, anti-metastatic

## Abstract

**Background:**

PG545 is a heparan sulfate (HS) mimetic that inhibits tumour angiogenesis by sequestering angiogenic growth factors in the extracellular matrix (ECM), thus limiting subsequent binding to receptors. Importantly, PG545 also inhibits heparanase, the only endoglycosidase which cleaves HS chains in the ECM. The aim of the study was to assess PG545 in various solid tumour and metastasis models.

**Methods:**

The anti-angiogenic, anti-tumour and anti-metastatic properties of PG545 were assessed using *in vivo* angiogenesis, solid tumour and metastasis models. Pharmacokinetic (PK) data were also generated in tumour-bearing mice to gain an understanding of optimal dosing schedules and regimens.

**Results:**

PG545 was shown to inhibit angiogenesis *in vivo* and induce anti-tumour or anti-metastatic effects in murine models of breast, prostate, liver, lung, colon, head and neck cancers and melanoma. Enhanced anti-tumour activity was also noted when used in combination with sorafenib in a liver cancer model. PK data revealed that the half-life of PG545 was relatively long, with pharmacologically relevant concentrations of radiolabeled PG545 observed in liver tumours.

**Conclusion:**

PG545 is a new anti-angiogenic clinical candidate for cancer therapy. The anti-metastatic property of PG545, likely due to the inhibition of heparanase, may prove to be a critical attribute as the compound enters phase I clinical trials.

Tumour angiogenesis has become a prominent drug target for cancer therapy (Carmeliet, 2005) and agents designed to target the vascular endothelial growth factor (VEGF) family and the fibroblast growth factor (FGF) family, along with their corresponding receptor tyrosine kinases, have received considerable attention (Sparano *et al*, 2004; Folkman, 2007). Anti-angiogenic therapies directed against these targets have resulted in promising and well-validated therapeutics, with both biologicals and small-molecule drugs that target VEGF/VEGFR already approved for a number of cancers. Nonetheless, most cancer deaths are due to the development of metastases (Eccles and Welch, 2007) and angiogenesis inhibitors do not necessarily affect this process. Indeed, recent pre-clinical studies have raised some concerns about the impact of such agents and metastatic disease (Ebos *et al*, 2009; Paez-Ribes *et al*, 2009; Steeg *et al*, 2009).

Cleavage of HS by the endo-*β*-glucuronidase heparanase (HPSE) is strongly implicated in cell dissemination associated with tumour metastasis (Barash *et al*, 2010). Endo-*β*--glucuronidase heparanase is also involved in angiogenesis, where it releases sequestered HS-binding angiogenic growth factors (GFs) from the extracellular matrix (ECM) (Nakajima *et al*, 1988; Vlodavsky *et al*, 1996; Zetter, 1998; Goldshmidt *et al*, 2002). It also induces VEGF expression (Zetser *et al*, 2006). The upregulation of HPSE in many tumours has been linked to poor prognosis (Chen *et al*, 2008; Mikami *et al*, 2008; Rivera *et al*, 2008; Zheng *et al*, 2009). The use of heparin, heparin derivatives, sulfated oligosaccharides and suramin and its analogues to inhibit HPSE activity have shown promise as anti-tumour or anti-metastatic agents (Vlodavsky *et al*, 1994; Marchetti *et al*, 2003; Basche *et al*, 2006; Norrby, 2006). The clinical utility of a mixture of sulfated oligosaccharides (PI-88) with dual heparanase and angiogenic inhibitory activity has been previously demonstrated in a phase II clinical trial for hepatocellular carcinoma (HCC) (Liu *et al*, 2009). The limited utility of current angiogenesis inhibitors and the critical need to address the metastatic component of cancer together provide a strong rationale to develop new HPSE inhibitors typified by aglycon-functionalised sulfated oligosaccharides (Karoli *et al*, 2005; Johnstone *et al*, 2010).

We report here the pharmacological profile of PG545, a synthetic, fully sulfated HS mimetic. We have previously demonstrated that PG545 is a potent heparanase inhibitor (*K*_i_=6 nM) and a potent angiogenesis inhibitor (⩽1 *μ*M) using endothelial cell assays *in vitro* and the *ex vivo* the rat aortic ring assay. Moreover, PG545 was found to have mild anti-coagulant properties using the activated partial thromboplastin time test and the Heptest compared with PI-88 or earlier PG500 series compounds (Dredge *et al*, 2010). In contrast to other HS mimetics which are mixtures, PG545 is a single molecular entity. PG545 induces potent anti-angiogenic activity *in vivo* together with broad acting pre-clinical anti-tumour and anti-metastatic efficacy with a pharmacokinetic (PK) profile to support less frequent dosing compared with other HS mimetics. An open-label, single centre phase I study of the safety and tolerability of PG545 in patients with advanced solid tumours commenced in late 2010 (http://www.clinicaltrials.gov).

## Materials and methods

### Compound synthesis and preparation

**Figure 1:**
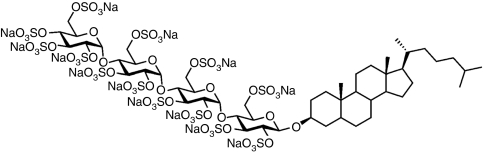
The structure of PG545.

PG545 is a fully sulfated tetrasaccharide functionalised with a cholestanyl aglycon (Figure 1) designed at Progen Pharmaceuticals Ltd (Brisbane, QLD, Australia). PG545 and radiolabeled PG545, the latter being synthesised from [1*α*,2*α*(n)-^3^H]-cholesterol (GE Healthcare Australia Pty. Ltd., Rydalmere, NSW, Australia), were manufactured at Progen Pharmaceuticals Ltd and PharmaSynth (Brisbane, Australia). The tyrosine kinase inhibitor (TKI) sorafenib is an inhibitor of Raf-1 and other kinases involved in neovascularisation and tumour progression (Wilhelm *et al*, 2004) indicated for renal cell carcinoma and HCC. It was approved by US FDA in 2007 for patients with unresectable HCC and is the first approved systemic drug therapy for liver cancer. Sorafenib was sourced from Bayer Health Care (Leverkusen, Germany). PG545 was dissolved using cell culture medium for *in vitro* or *ex vivo* experiments and phosphate buffered saline (PBS), pH 7.2 for *in vivo* studies.

### Cell lines and reagents

LL/2, Hep3b2.1-7 and HT-29 tumour cell lines were obtained from the American Type Culture Collection and working stocks did not exceed four passages. MDA-MB-231, PC3, Cal27 and HepG2 were from laboratory stocks at the Diamantina Institute, Brisbane, Australia. Identity was verified by STR analysis conducted by Cell Bank Australia (Sydney, Australia) in February 2010. B16F0 was a gift from Dr Glen Boyle (Queensland Institute of Medical Research, Brisbane, Australia). Hep3b2.1-7 and PC3 were cultured in RPMI-1640 (Invitrogen, Mt Waverley, Australia) while MDA-MB-231, B16F0, HT-29, HepG2, Cal27 and LL/2 were cultured using DMEM (Invitrogen) and supplemented with 10% fetal bovine serum (Invitrogen or Hyclone, Logan, UT, USA), 100 IU ml^−1^ penicillin (Invitrogen), 100 *μ*g ml^−1^ streptomycin (Sigma-Aldrich, Castle Hill, Australia), 1 mM sodium pyruvate (for LL/2 and HepG2 only: Invitrogen), 25 mM HEPES (HepG2: Invitrogen) and 0.4 mg ml^−1^ hydrocortisone (Cal27: Sigma-Aldrich).

### Animals

Athymic (nude), SCID, FvB, BALB/c and C57Bl6 mice were obtained from the Animal Resources Centre (Perth, Australia). All animal studies were approved by the University of Queensland or the University of Adelaide Animal Ethics Committees. During the studies, the care and use of animals was conducted in accordance with the principles outlined in the Australian Code of Practice for the Care and Use of Animals for Scientific Purposes, 7th Edition, 2004 (National Health and Medical Research Council). For the HT-29 study, the protocol was approved by the Institutional Animal Care and Use Committee and the study was performed at the AAALAC certified animal facility in Pharma Legacy (Shanghai, China).

### *In vivo* angiogenesis

Angiogenesis *in vivo* was evaluated using the Angiosponge model in which subcutaneously implanted, agarose-captured FGF2 gelfoam sterile sponges (Pfizer, West Ryde, Australia) induces vascularisation over a 10-day period responsive to angiogenesis inhibition as determined by the number of vessels with identifiable lumen that stained positive for CD31/PECAM-1, an antigen present specifically on endothelial cells (McCarty *et al*, 2002). PG545 was administered s.c. on the flank distal to the AngioSponge implant. This method was also used to examine five medium-sized primary tumours from each group of Lewis Lung Carcinoma (LL/2)-bearing mice.

### Tumour models and drug administration

In the orthotopic Hep3b2.1-7 human liver cancer model, treatment of mice (*n*=10) began 14 days after inoculation of 2.5 × 10^6^ cells. Mice received PG545 (or [^3^H]-PG545 for the efficacy/PK study) using either a once- or twice-weekly dosing schedule. To initiate tumour xenografts, MDA-MB-231, PC3, Cal27 and HepG2 cells were injected s.c. into nude mice (*n*=8) at 4, 5, 2 and 2 × 10^7^ cells ml^−1^, respectively. PG545 was injected s.c. in a volume of 100–200 *μ*l PBS once the average tumour volume was ∼100–200 mm^3^. In the B16F0 lung metastasis model, 2 × 10^5^ cells were injected via the tail vein of C57Bl6 mice (*n*=8). In the syngeneic LL/2 model, single dose or weekly treatments with PG545 at 20 or 40 mg kg^−1^ and sorafenib daily at 60 mg kg^−1^ commenced on the day of inoculation with 2 × 10^6^ cells. Surface lung macrometastases were counted for each group (*n*=9) upon study termination. In the intrasplenic HT-29 xenograft model, 1 × 10^6^ cells in 100 *μ*l were injected into the spleen. The next day, PG545 (20 mg kg^−1^) was administered to mice (*n*=12) each week (qw) over 8 weeks and surface liver and colon metastatic nodules were counted for each group (*n*=12) upon study termination. Solid tumours were measured in two dimensions (length and width) and the tumour volume (cm^3^) was calculated using the equation: *V*=length × width^2^ × *π*/6. The anti-tumour activity was expressed as percent tumour growth inhibition (%TGI). Further details are available in Supplementary Methods.

### PK sampling and analysis

Levels of [^3^H]-PG545 determined in blood and tumour samples collected at 12, 24, 48 and 96 h post treatment and levels of PG545 in plasma samples (LC–MS/MS method) collected at 2, 4, 8, 24, 48, 72 and 96 h post treatment were determined as outlined in Supplementary Methods. The PK parameters, *C*_max_ (maximum plasma concentration), *T*_max_ (time of maximum plasma concentration), *k*_elim_ (terminal elimination rate constant), *t*_½_ (half-life) and AUC (area under the concentration *vs* time curve) were derived from the blood [^3^H]-PG545 or plasma PG545 concentration *vs* time data using model independent methods. Analysis of the tumour [^3^H]-PG545 concentration *vs* time data required use of a more physiologically based PK model such that tumour concentrations were estimated from the tumour mass being perfused with blood containing [^3^H]-PG545 and where the blood [^3^H]-PG545 concentration changed over time.

### Statistics

For multiple comparisons, a one-way analysis of variance (ANOVA) followed by a *post hoc* Holm-Sidak test or a Dunnett's test was used, provided the data passed a normality test or the equal variance test. For comparison between a control and a treatment group, an unpaired *t*-test was used. A *P*-value <0.05 was considered significant.

## Results

### PG545 inhibits angiogenesis *in vivo*

**Figure 2:**
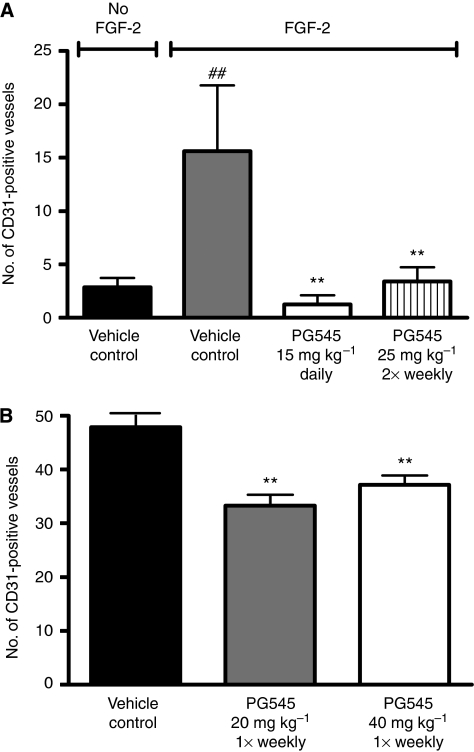
PG545 inhibits angiogenesis *in vivo*. (**A**) PG545 treatment was shown to possess anti-angiogenic activity in the AngioSponge model following evaluation of CD31/PECAM-1 positive endothelial cells after immunohistochemical staining of blood vessels. ^##^*P*<0.01 vs vehicle control without FGF-2; ^**^P<0.01 *vs* vehicle control and FGF-2. (**B**) Treatment with PG545 at 20 and 40 mg kg^−1^ once-weekly (s.c.) resulted in a significant (*P*<0.001) decrease in CD31 endothelial staining in tumours by 30 and 22%, respectively.

In the AngioSponge model, PG545 was administered either daily or twice-weekly. Significant inhibition of CD31 staining was observed (Figure 2A). As the duration of the AngioSponge model is 10 days in total, once-weekly treatment was not examined in this model. However, the effect of once-weekly dosing with PG545 on tumour angiogenesis was determined in tumour-bearing mice (LL/2 model). Assessment of CD31-positively stained vessels with identifiable lumen in the primary (subcutaneous) tumours of mice confirmed that once-weekly treatment with PG545 significantly inhibited angiogenesis (Figure 2B).

### PK profile of radiolabeled PG545 indicates a long half-life in blood with evidence that efficacious doses penetrate solid tumours

**Figure 3:**
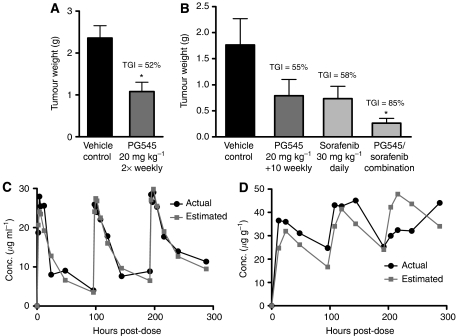
The anti-tumour activity of PG545 in the orthotopic HCC model Hep3b2.1-7 can be enhanced by addition of sorafenib and exhibits favourable PK profiles in blood and tumour tissue. (**A**) Efficacy of PG545 when administered at 20 mg kg^−1^ twice-weekly. (**B**) Efficacy of PG545 alone and in combination with sorafenib, when administered using a loading dose of 20 mg kg^−1^ followed by a twice-weekly maintenance dose of 10 mg kg^−1^. (**C**) Actual and estimated mean concentration *vs* time curve data for [^3^H]-PG545 in blood following administration of three bolus doses of [^3^H]-PG545 at 20 mg kg^−1^ at 96 h intervals. (**D**) Actual and estimated mean concentration *vs* time curve data in tumour tissue of mice treated with three doses of [^3^H-PG545] at 20 mg kg^−1^, 96 h apart. ^*^P<0.05 *vs* vehicle control (unpaired *t*-test or ANOVA followed by Dunnett's test).

PG545 significantly reduced solid tumour growth in the orthotopic liver cancer model Hep3b2.1-7 under two different regimens: a twice-weekly s.c. administration schedule at 20 mg kg^−1^ (Figure 3A) or using 20 mg kg^−1^ as a loading dose followed by a twice-weekly 10 mg kg^−1^ maintenance dose (Figure 3B). For compounds with long half-lives, a loading dose is often used to quickly increase the plasma concentration of a drug to the desired steady state levels (Paw, 2006). These dosing schedules resulted in body weight loss of 9% in the 20 mg kg^−1^ twice-weekly group and 4% in the 20 mg kg^−1^ followed by twice-weekly 10 mg kg^−1^ group (data not shown). The data generated in separate experiments using 20 mg kg^−1^ twice-weekly or as a loading dose (followed by 10 mg kg^−1^ as a maintenance dose) exhibited a similar efficacy profile (Tumour Growth inhibition (TGI) values of 52% versus 55%). In combination, twice-weekly dosing with PG545 and sorafenib (30 mg kg^−1^, qd) led to a statistically significant reduction in tumour weight compared with control (TGI=85%). Once-weekly dosing with PG545 at 20 mg kg^−1^ followed by 10 mg kg^−1^ increased the TGI to 65% compared to the twice-weekly regimen but the enhanced anti-tumour effects observed in combination with sorafenib were not improved with once-weekly dosing (data not shown).

The PK profile of PG545 in tumour-bearing animals was determined using 20 mg kg^−1^ [^3^H]-PG545 administered s.c. every 96 h for a total of three doses. The concentration of PG545 was assessed in both blood and tumour samples. The PK profile in blood indicated that the mean elimination half-life (*t*_½_) of [^3^H]-PG545 was >50 h for mice administered up to three s.c. bolus doses at 96 h intervals (Table 1). Visual inspection of the mean blood [^3^H]-PG545 concentrations *vs* time curve data showed that the mean blood *C*_max_ values were in the range 25.9–29.0 *μ*g ml^−1^ and the corresponding mean *T*_max_ values were in the range 2–6 h (Figure 3C).

Visual inspection of the mean tumour [^3^H]-PG545 concentration *vs* time curve data showed that the tumour *C*_max_ values were in the range 36.6–45.1 *μ*g g^−1^ and that the corresponding mean *T*_max_ values in the tumour tissue were 12 h (one dose), 48 h (two doses) and ⩾96 h (three doses) (Figure 3D). Using a one-compartment PK model, the estimated mean rate constant for uptake of [^3^H]-PG545 from the bloodstream into the tumour (*t*_½b_), derived from the mean blood and tumour concentration *vs* time data, was in the range 12.8–16.5 h. The corresponding estimated mean *t*_½_ for return of [^3^H]-PG545 from the tumour into the bloodstream (*t*_½t_), derived from the mean blood and tumour concentration *vs* time data, was in the range 15.7–16.3 h (Supplementary data, Supplementary Table S1). The *C*_max_ values in liver and kidney reached 106 and 39 *μ*g g^−1^, respectively, following a single dose (data not shown). This equates to a tumour:liver ratio of 0.4 and a tumour:kidney ratio of 1.3, 12 h after a single dose, suggesting good distribution into tumour tissue compared with other organs. As a comparison, gefitinib (Iressa, Astra Zeneca, London, UK) has a tumour:liver ratio of 0.15 or 0.18 and a tumour:kidney ratio of 0.35 or 0.56, 2 h or 8 h, respectively, after a single dose in LoVo tumour xenografts (McKillop *et al*, 2005).

### PG545 inhibits tumour progression in a variety of xenograft models

**Figure 4:**
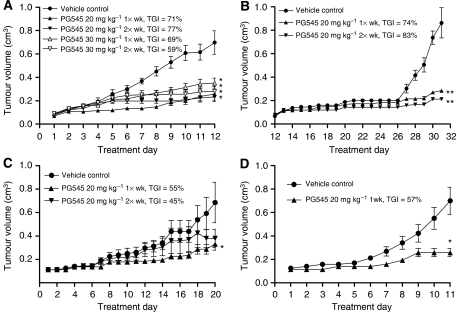
PG545 significantly inhibits solid tumour progression in (**A**) breast (MDA-MB-231), (**B**) prostate (PC3), (**C**) liver (HepG2) and (**D**) head and neck (Cal27) cancer models. Treatment commenced once tumours reached 100–200 mm^3^. ^*^P<0.05 *vs* vehicle control, ^**^P<0.01 *vs* vehicle control (repeated measures ANOVA followed by Dunnett's *post hoc* test, *n*=8–10 per group depending on study). TGI values are also shown.

Further investigation of once- or twice-weekly s.c. dosing schedules was performed in a variety of xenograft or syngeneic cancer models (Figure 4). Tumour growth inhibition in the MDA-MB-231 model (Figure 4A) was maximal at 20 mg kg^−1^ once-weekly (77%) or 20 mg kg^−1^ twice-weekly (71%) with 30 mg kg^−1^ groups inhibiting tumour growth by 69% (30 mg kg^−1^, once-weekly) or 59% (30 mg kg^−1^, twice-weekly). Body weight loss at the end of the study was 4% for 20 mg kg^−1^ once-weekly, 6% for 20 mg kg^−1^ twice-weekly, 11% for 30 mg kg^−1^ once-weekly and 10% for 30 mg kg^−1^ twice-weekly (data not shown). Employing the 20 mg kg^−1^ dose only in subsequent models, the differences noted between once- and twice-weekly schedules were not significant in the PC3 or HepG2 models. For example, in the PC3 model, once-weekly dosing led to a TGI of 74% while twice-weekly dosing resulted in a TGI of 83% (Figure 4B). Although body weight loss at 20 mg kg^−1^ once-weekly was 6%, the twice-weekly regimen was 16% (data not shown). However, in the HepG2 model (Figure 4C), the once-weekly (TGI=55%), but not twice-weekly (TGI=45%) schedule, significantly inhibited tumour growth to a similar extent as 20 mg kg^−1^ once-weekly treatment in the Cal27 model which produced a TGI of 57% (Figure 4D). In both models, 20 mg kg^−1^ once-weekly (and twice-weekly in the HepG2 model) led to body weight loss between 2% and 4% (data not shown). PG545 also significantly reduced primary tumour growth in the HT-29 model (Supplementary Figure S1a). The concentration of PG545 that is lethal to 50% of cells (LC_50_) using HUVEC (78 *μ*M), B16 (>200 *μ*M) or HepG2 cells (>100 *μ*M), indicated that PG545 is not cytotoxic *in vitro* (data not shown).

### PG545 induces potent anti-metastatic effects in experimental and spontaneous models of metastasis

**Figure 5:**
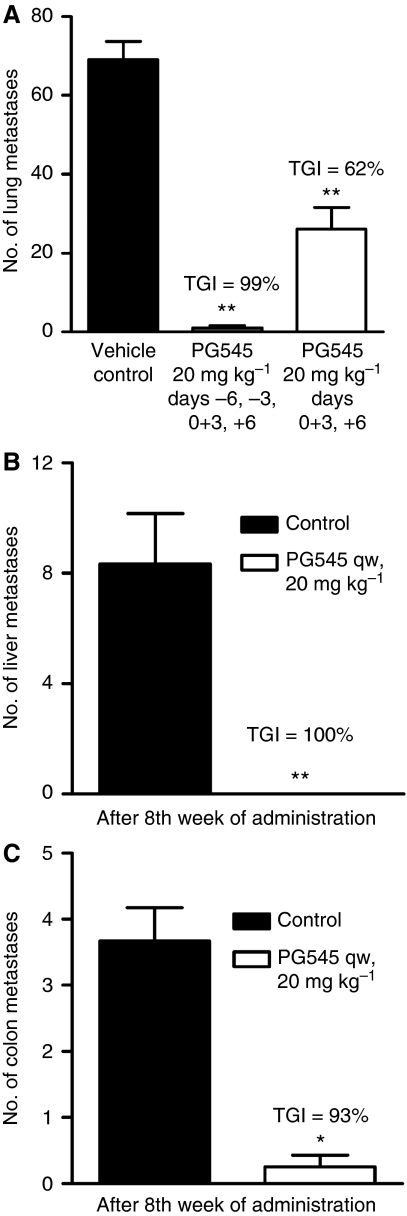
PG545 significantly inhibits metastases in the B16 experimental metastasis model for melanoma and the spontaneous metastasis model HT-29 (colon). (**A**) PG545 administered on days indicated either before or after tumour cell inoculation (Day 0) reduced metastatic development in the lungs of mice in the B16 model. (**B**) PG545 treatment once-weekly over 8 weeks completely prevented metastasis to the liver in the HT-29 model. (**C**) PG545 treatment once-weekly over 8 weeks significantly reduced metastasis to the colon in the HT-29 model. Data are presented as mean number of lung, liver or colon metastases with s.e. ^*^P<0.05, ^**^P<0.01 *vs* vehicle control (one-way ANOVA followed by Dunnett's test for B16 model or *t*-test for HT-29 model).

Twice-weekly injections of PG545 significantly inhibited the formation of metastases in the B16F0 experimental lung metastasis model, when administered either before or on the day of tumour cell inoculation (Figure 5A). When administered as a once-weekly schedule, PG545 also significantly inhibited the occurrence of spontaneously arising liver metastases (Figure 5B; Supplementary Figure S1b) and colon metastases (Figure 5C) in the HT-29 colon model. In terms of body weight loss, the nadir was 8% in the B16 model and 12% in the HT-29 model (data not shown).

**Figure 6:**
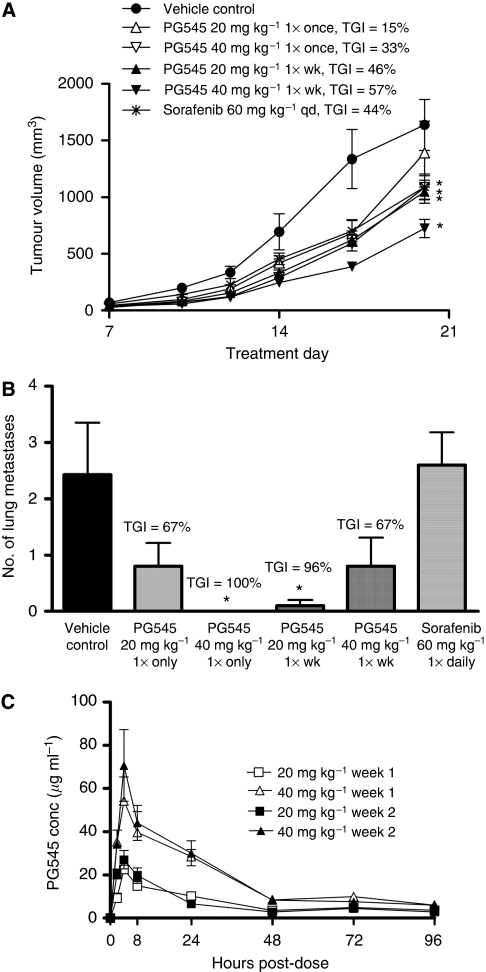
PG545 significantly inhibits solid tumour progression and spontaneous metastasis in the Lewis Lung Carcinoma model (LL/2). (**A**) PG545 treatment once-weekly (total 3 weeks) significantly inhibited solid tumour growth. (**B**) Either a single injection or weekly dosing inhibited metastasis, which was significant in the 20 mg kg^−1^ 1 × week group and the 40 mg kg^−1^ single dose group, whereas sorafenib had no effect on metastasis. Data are presented as mean number of lung metastases with s.e. (**C**) PG545 plasma concentration *vs* time curves following a once-weekly dose of PG545 in week 1 and week 2. ^*^P<0.05 *vs* vehicle control (one-way ANOVA followed by Holm-Sidak test or Dunnett's test).

The Lewis Lung Carcinoma model (LL/2) provided another model to investigate the effect of PG545 on spontaneous metastasis. PG545 was administered at weekly intervals or in some cases, as a single injection from the day of tumour inoculation. The body weight nadir during the study constituted a loss of 5% (data not shown). In addition to significant inhibition of primary tumour growth (Figure 6A), PG545 significantly reduced the number of lung metastases, with complete abrogation in the single dose 40 mg kg^−1^ group and 96% inhibition in the 20 mg kg^−1^ once-weekly group (Figure 6B). However, it is not clear why 40 mg kg^−1^ once-weekly failed to reach significance but interestingly sorafenib demonstrated no effect on metastasis in this model. Plasma concentration *vs* time curves indicated that efficacious exposure levels should be between 20 and 70 *μ*g ml^−1^ (Figure 6C) and PK analysis revealed the apparent *t*_½_ to range between 28 and 39 h with a *C*_max_, *T*_max_ and to a lesser extent, an AUC, comparable to that observed in the liver cancer model using the radiolabeled compound (Table 2). The difference between the half-life estimation of [^3^H]-PG545 and PG545 by LC/MS/MS may be explained by experimental variations/matrices and the possibility that the radiolabel was no longer attached to the parent molecule, thus estimating the *t*_½_ for removal of a metabolite to which the label is attached.

## Discussion

Heparanase-mediated degradation of HS is involved in tumour angiogenesis and metastasis, making it a major target for cancer research (Hammond *et al*, 2006; McKenzie, 2007; Barash *et al*, 2010). Clinical studies have typically noted a poorer prognosis in patients with high expression of heparanase, presumably due to its association with an aggressive, metastatic phenotype (Chen *et al*, 2008; Mikami *et al*, 2008; Rivera *et al*, 2008; Zheng *et al*, 2009). The pivotal role of VEGF and its receptors in promoting angiogenesis is also well established, and both antibody and small-molecule inhibitors of VEGF signaling have shown clinical utility. Despite no specific FGF inhibitors being clinically available, their relevance as a cancer therapy has become increasingly clear due to the increased expression of FGF family members in late-stage tumours following phenotypic resistance to VEGFR2 blockade (Casanovas *et al*, 2005). We previously identified a series of HS mimetics, of which PG545 was one, as potent inhibitors of heparanase, GFs including VEGF and FGFs and *ex vivo* angiogenesis (Dredge *et al*, 2010). Herein, we extend those findings to the pre-clinical arena to demonstrate the *in vivo* potency of PG545 in angiogenesis, solid tumour and metastasis models and provide initial data on the pharmacokinetics associated with the compound.

The AngioSponge model highlighted that daily and twice-weekly dosing with PG545 induced a potent anti-angiogenic effect *in vivo*, as measured by CD31 expression. In the LL/2 model, decreases in CD31 expression were also statistically significant using once-weekly dosing. Unfortunately, as only five representative medium-sized tumours from each group were available, any dose-dependent correlation between reduction in CD31 staining and inhibition of solid tumour growth is not apparent. Overall, the data confirmed that a once- or twice-weekly dosing schedule is anti-angiogenic *in vivo* and this schedule is less frequent compared with those of other heparin-derived products or HS mimetics (Gandhi and Mancera, 2010). The body weight loss in mice may be species-specific because PG545 is not cytotoxic *in vitro* or *in vivo* (vacuolation was most common pathology finding) and did not lead to significant body weight loss in other species during toxicity studies (data not shown). The absence of bruising in PG545-treated nude mice illustrates the mild anti-coagulant properties of PG545 (Dredge *et al*, 2010). Although high doses of PG545 increased activated partial thromboplastin time (which was reversible) in toxicology studies, it is not clear whether this is due to a depletion of clotting factors or a mild anti-coagulant effect (data not shown).

PK data were generated using radiolabeled PG545 administered to satellite groups of tumour-bearing mice (Hep3b2.1-7). A relatively long half-life (*t*_½_) in blood (>50 h) was observed. The concentrations of [^3^H]-PG545 measured within the liver tumours of mice confirmed entry into tumour tissue. A one-compartment PK model found each of the estimated parameter values was internally consistent following administration of one, two or three doses (Supplementary data; Supplementary Table S1). However, visual inspection of the graph for tumour-bearing mice administered three doses of [^3^H]-PG545, with each dose separated by a 96-h interval, showed that the tumour concentration *vs* time curve for the third dose is different from that for the preceding two doses. The reason for this is unclear though it is possible that PG545 could have induced biological change within the tumour vasculature – such an assumption would not necessarily be applicable to the one-compartment PK model.

Tumour growth in the Hep3b2.1-7 model was significantly reduced following the once- and twice-weekly dosing schedule for PG545 or using a loading and maintenance dose approach, to an extent similar to that seen following treatment with sorafenib, a TKI approved for HCC. The combination of PG545 and sorafenib produced a TGI up to 85%, thus illustrating some potential utility for combination modalities using PG545. Taken together with the PK profile, the efficacy data indicated that once-weekly dosing may provide an optimal dosing frequency for PG545. This was further confirmed using a variety of subcutaneously implanted xenograft or syngeneic models of prostate, breast, liver and head and neck cancer in the mouse.

We next tested the anti-metastatic activity of PG545 in three murine models of metastasis. In the B16 melanoma model, short-term therapy with PG545 was initiated either before or immediately after i.v. tumour inoculation of B16 cells and confirmed that both schedules significantly inhibited the development of lung metastases. In the spontaneously metastasising HT-29 colon model, complete abrogation of liver metastases was achieved using a weekly regimen of 20 mg kg^−1^ PG545 over 8 weeks (a dose in week 6 was omitted due to body weight concerns). In the spontaneously metastasising Lewis Lung Carcinoma model, PG545 significantly reduced the number of metastases and PK data estimated the efficacious plasma concentration range starts at ∼20 *μ*g ml^−1^. This potent anti-metastatic effect of PG545 may differentiate it from other angiogenesis inhibitors, which have been associated with an acceleration of metastasis under certain circumstances (Ebos *et al*, 2009; Paez-Ribes *et al*, 2009). It is hypothesised that a metastatic ‘conditioning’ effect may occur via multiple mechanisms following treatment with angiogenesis inhibitors (Ebos *et al*, 2009; Paez-Ribes *et al*, 2009) which posed the question – are there any alternative anti-angiogenic agents that might not evoke this malignant behaviour? (Loges *et al*, 2009). Thus, further elucidation of the contrasting mechanisms of action and possible differences in efficacy between HS mimetics and TKI/biologics is warranted.

In conclusion, we have clearly demonstrated the anti-tumour and anti-metastatic properties of PG545, which to our knowledge is the first HS mimetic that is a single molecular entity to emerge as a clinical candidate. In addition to its anti-angiogenic properties, it is the anti-heparanase/anti-metastatic activity that may differentiate PG545 from other anti-angiogenic agents. Particularly in light of recent preclinical findings relating primarily to anti-VEGF therapies, this property may become a critical attribute as PG545 progresses towards the clinic.

## Figures and Tables

**Table 1:** Model independent pharmacokinetic analysis of blood [^3^H]-PG545 in tumour-bearing mice (Hep3b model) administered at 20 mg kg^−1^ every 96 h

**Parameter**	**First dose**	**Second dose**	**Third dose**
*C*_max_ (*μ*g ml^−1^)	28	26	29
*T*_max_ (h)	4	2	6
*t*_½_ (h)	65	ND	117
AUC_0−t_ (*μ*g.h ml^−1^)	991	1208	1556

Abbreviation: AUC=area under the concentration *vs* time curve.

Note: *t*_½_ after second dose could not be determined (ND) as blood concentrations for at least four half-lives are required to provide accurate estimates of elimination rate constant.

**Table 2:** Model independent pharmacokinetic analysis of PG545 in the plasma of tumour-bearing mice (Lewis Lung Carcinoma)

	**Week 1**			**Week 2**		
**Parameter**	**20 mg kg^−1^**	**40 mg kg^−1^**	**60 mg kg^−1^**	**20 mg kg^−1^**	**40 mg kg^−1^**	**60 mg kg^−1^**
*C*_max_ (*μ*g ml^−1^)	23	55	79	27	71	94
*T*_max_ (h)	5	3	5	4	4	4
*t*_½_ (h)	38	32	39	31	28	35
AUC_0−t_ (*μ*g.h ml^−1^)	687	1713	2666	663	1781	3056

Abbreviation: AUC=area under the concentration *vs* time curve.
