# Supplementary figures and images for: PG545, a dual heparanase and angiogenesis inhibitor, induces potent anti-tumour and anti-metastatic efficacy in preclinical models
Source: Br J Cancer. 2011 Feb 1;104(4):635–42. doi: 10.1038/bjc.2011.11 (PMC3049593; doi:10.1038/bjc.2011.11)

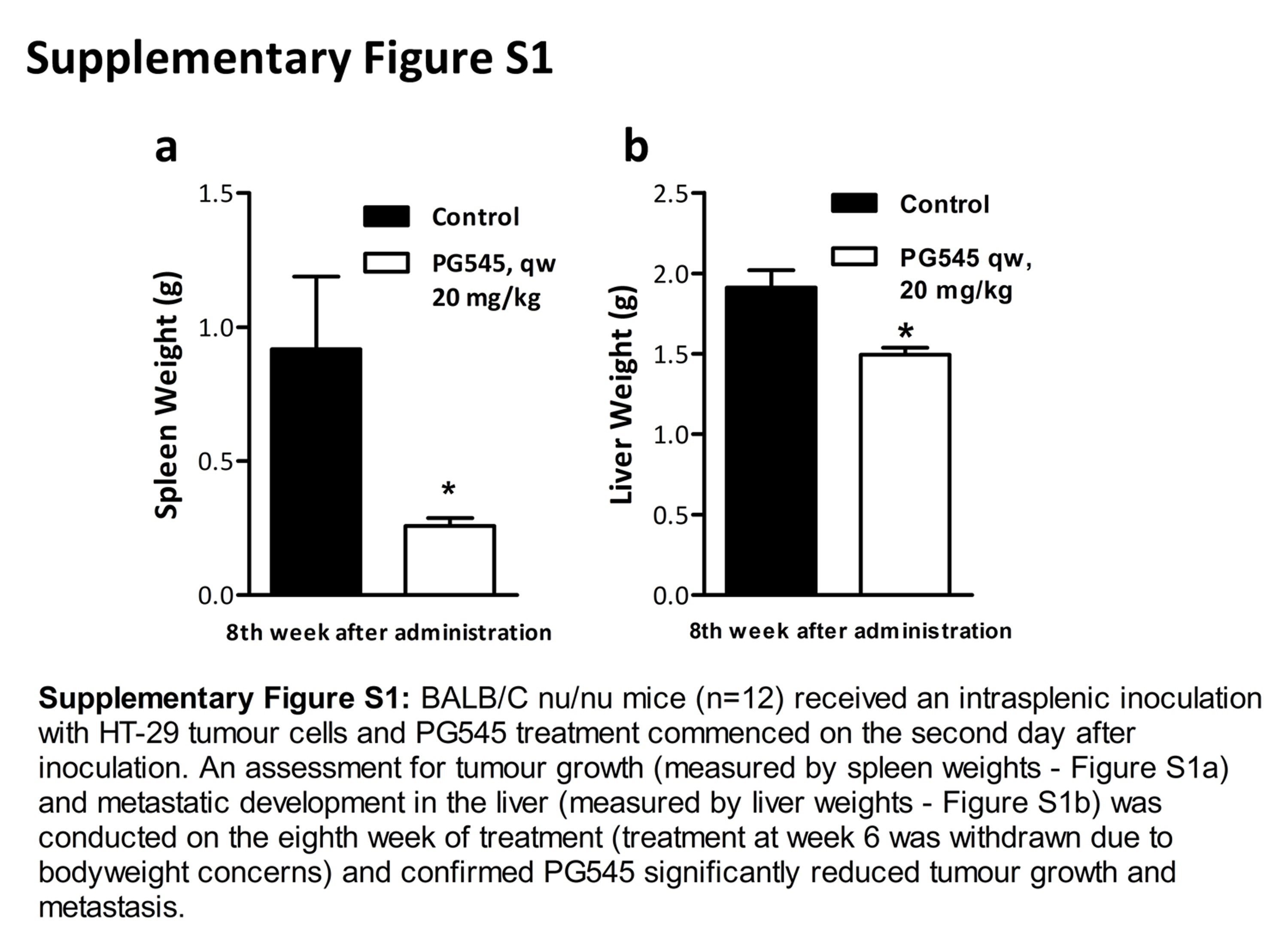

Supplement: Supplementary Figure S1 [file bjc201111x1.tif]
