# Supplementary material for: PG545, a dual heparanase and angiogenesis inhibitor, induces potent anti-tumour and anti-metastatic efficacy in preclinical models
Source: Br J Cancer. 2011 Feb 1;104(4):635–42. doi: 10.1038/bjc.2011.11 (PMC3049593; doi:10.1038/bjc.2011.11)
Supplement: Supplementary Table S1 [file bjc201111x2.pdf]

Table S1 – Pharmacokinetic analysis of mean tumour [^{3}H]-PG545 concentrations in mice

Model Independent Analysis of Mean Tumor [^{3}H]-PG545 Conc. Versus Time Data

Parameter	First Dose	Second Dose	Third Dose	Notes: C_{max} and T_{max} were derived from visual inspection of the data following s.c. administration of up to three doses of [^{3}H]-PG545 at 96 h intervals
C_{max} (μg/ml)	37	45	44	
T_{max} (h)	12	48	96	
$AUC_{0\text{-}t}$ (μg.h/g)	2800	3662	3304	

One Compartment Model derived from Mean Tumor [^{3}H]-PG545 Conc. Versus Time Data

Parameter	First Dose	Second Dose	Third Dose	Notes: Based on assumptions including -
K_bV/M	0.1463	0.1409	0.1131	1) All processes are linear with no saturation or rate limiting effects and occur at a single point with no spatial distribution effects.<br>2) The presence of PG545 does not induce biological change within the timeframe of the experiment.<br>3) Tumor [^{3}H]-PG545 conc is driven by the blood conc of [^{3}H]-PG545<br>4) Rate of uptake is proportional to blood conc.<br>5) Rate of return from tumor to blood is proportional to tumor conc.
K_b (h^{-1})	0.0541	0.0522	0.0419	
$t_{½}b$ (h)	12.8	13.3	16.5	
K_t (h^{-1})	0.0441	0.0449	0.0425	
$t_{½}t$ (h)	15.7	15.4	16.3	

where
K_b is the rate constant of uptake into the tumor, in h^{-1}
K_t is the rate constant of uptake from the tumor, in h^{-1}
V is the mean total volume of circulating blood in an adult male mouse, in ml
M is the mean mass of the tumor in mice included in this study, in g
$t_{½}b$ the estimated mean rate constant for uptake of [3H]-PG545 from the bloodstream into the tumor
$t_{½}t$ the estimated mean rate constant for uptake of [^{3}H]-PG545 from the tumor back to the bloodstream
