# Supplementary material for: PG545, a dual heparanase and angiogenesis inhibitor, induces potent anti-tumour and anti-metastatic efficacy in preclinical models
Source: Br J Cancer. 2011 Feb 1;104(4):635–42. doi: 10.1038/bjc.2011.11 (PMC3049593; doi:10.1038/bjc.2011.11)
Supplement: Supplementary Methods [file bjc201111x3.pdf]

Supplementary Methods

Cell lines, cell culture, reagents and compounds

Tumour cell lines were cultured in RPMI1640 or DMEM cell culture medium (Invitrogen, Mt Waverley, Australia), 10% foetal bovine serum (Invitrogen or Hyclone/Quantum Scientific), 100 IU/mL penicillin (Invitrogen), 100 µg/mL streptomycin (Invitrogen or Sigma-Aldrich, Castle Hill, Australia). Hep3b2.1-7 human liver cancer cells, HT-29 human colon cancer cells and LL/2 mouse lung cancer cells were sourced from American Type Culture Collection (ATCC) (Rockville, MD, USA) and working stocks did not exceed P4. The human breast carcinoma cell line MDA-MB-231, prostate PC3, Cal27 and HepG2 cell lines were from laboratory stocks at Diamantina Institute, Brisbane. Identity was verified by STR analysis conducted by Cell Bank Australia in February 2010. B16F0 murine melanoma cells were obtained from Dr Glen Boyle (Queensland Institute of Medical Research, Brisbane, Australia).

Sorafenib, used in experiments conducted by vivoPharm, was manufactured and supplied by Bayer Health Care AG (Leverkusen, Germany). Radiolabeled PG545 was synthesized from [1α,2α (n)-^{3}H]-cholesterol (GE Healthcare).

AngioSponge™ Model

Gelfoam sponges were cut into approximately 5 x 5 x 7 mm pieces under sterile conditions and pre-soaked overnight with sterile PBS. As the growth factor, FGF-2, is known to be involved in inducing formation of new blood vessels (angiogenesis), it was omitted from sponges used for Group 1. Consequently, Group 1 served as the negative (baseline) control group, which should not exhibit extensive capillary formation within the implant during the study period. FGF-2 was diluted to a final concentration of 4 µg/mL in 1.6% agarose and added to the sponges for treatment groups. The Gelfoam pieces were placed in the growth factor solution and mixed by gentle swirling; the suspension was then placed in a Petri dish to allow polymerisation to take place. The Gelfoam sponges (containing agarose, with or without growth factors) are the AngioSponges™.

The vehicle control (NMP:PEG300 (1:9, v/v)) was administered by oral gavage (p.o.) once daily. PG545 was administered by subcutaneous (s.c.) injection either once daily (15 mg/kg or twice weekly (25 mg/kg). Sorafenib was administered by oral gavage (p.o.) once daily (60mg/kg, Group 6). Animals were treated over a 10 day period, starting at Day 0 immediately after implantation of the AngioSponge™. Twenty-four hours following the final treatment (Day 10), animals were euthanised by CO_2 inhalation and the AngioSponge™ recovered. The vascularised sponge was embedded in OCT compound and snap-frozen in liquid nitrogen in preparation for CD31/PECAM-1 immunohistochemistry.

Vascularised AngioSponge™ frozen samples were cryo-sectioned (6 µm thickness) and stained with a monoclonal rat anti-mouse CD31 and a secondary anti-rat secondary

antibody. Visualisation was achieved using streptavidin peroxidase and 3,3'-diaminobenzidine and the sections were also rinsed with water and counter-stained with Lillie-Mayers haematoxylin. A CD31 positive control slide showed a consistent vascular staining pattern throughout. A negative control slide showed a complete absence of staining. For quantification, three independent visual fields (20x magnification) were chosen and positive stained spots or vessels with identifiable lumen were counted. Angiogenesis inhibition and induction were calculated using the equations:

$$\% \text{ Inhibition} = [(A-B)/A \times 100]$$

$$\% \text{ Induction} = [(B-C)/B \times 100]$$

where A is the mean CD31 count from the mice with implanted sponges containing growth factor and treated with Vehicle Control.

B is the mean CD31 count from the mice with implanted sponges containing growth factor and treated with PG545 or sorafenib.

and C is the mean CD31 count from the mice with implanted sponges lacking growth factor and treated with Vehicle Control.

Tumour models

For the xenograft models MDA-MB-231, HepG2, Cal 27 or PC3 cells were resuspended in cold PBS:Matrigel™ (1:1, v/v) to final concentrations of 4-, 5, 2 and 2 x 10^7 cells/ml, respectively. Then, 100 µL was injected subcutaneously into the flank of each anaesthetized nude mouse except for Cal27 in which 50 µL was injected. PG545 was injected s.c. in a volume of 100 µL PBS once the average tumour volume was approximately 100-200 mm^3 as indicated in the figure legends.

In the Hep3b2.1-7 model, the cells resuspended in HBSS:Matrigel™ (1:1, v/v) to a final concentration of 5 x 10^8 cells/mL were inoculated following an incision into the skin directly over the liver to expose the main lobe of the liver. The needle was introduced into the main lobe of the liver, where 2.5 x 10^6 Hep3b2.1-7 cells (in 5 µL with 50% Matrigel) were discharged. The treatment of mice began 24 days after inoculation of the Hep3B2.1-7 cells. Mice received PG545 (or [^{3}H]-PG545 for the efficacy/PK study) using either a once or twice weekly dosing schedule (see figure legends for dose schedules in each study) for up to three weeks. Mice received PG545 (or [^{3}H]-PG545 for the efficacy/PK study) using either a once or twice weekly dosing schedule (see figure legends for dose schedules in each study) for up to three weeks.

In the B16 experimental metastasis study, PG545 was administered as a twice weekly regimen both before and after tumour cell inoculation (days -6, -3, 0, +3, +6) or only after tumour cell inoculation (days 0, +3, +6). B16 cell (2x10^5) were inoculated via tail vein injections and mice were culled and assessed for lung metastases on day 12.

In the LL/2 mouse model, treatment of mice commenced on the day of inoculation (Day 0). Mice inoculated subcutaneously were treated with either Vehicle Control (NMP:PEG300 (1:9, v/v), once daily, p.o., PG545 (20 or 40 mg/kg, each once only or

once weekly or Sorafenib (60mg/kg, once daily, p.o.). The treatments were continued for three weeks. The lungs were excised from all mice at the time of culling for metastasis count determination. Surface lung macrometastases were counted for each group upon study termination.

In the HT-29 model, the lumbar region skin was sterilized with 5% tincture and 75% ethanol, respectively. One-cm-long incision was made to expose the spleen before 1×10^6 cells in 100 μL was injected into the spleen. The incision was then closed in layers with single interrupted suture and then cleaned with 75% ethanol. The next day, PG545 (20 mg/kg) was administered to mice (n=12) each week (qw) over 8 weeks and surface liver and colon metastatic nodules were counted for each group (n=12) upon study termination. Spleen and liver weights were also measured as indirect assessment of tumour burden.

In relevant models, solid tumours were measured in two dimensions (length and width) and the tumour volume (mm^3) calculated using the equation $V = length \times width^2 \times \pi/6$.The anti-tumour activity was assessed by the comparison of the mean tumour volume of the treated groups to the mean tumour volume of the control group and expressed as the percent Tumour Growth Inhibition (TGI) using the equation:

$$\%TGI = (1-((V_{endPG545} - V_{startPG545}) / (V_{endControl} - V_{startControl})))*100$$

$V_{endPG545}$ = Ending Tumour Volume of PG545-treated animal
$V_{startPG545}$ = Starting Tumour Volume of PG545-treated animal
$V_{endControl}$ = Ending Tumour Volume of Control animal
$V_{startControl}$ = Starting Tumour Volume of Control animal

Pharmacokinetic sampling and analysis

Samples were collected from three animals at each of four time points – 12 hours, 24 hours, 48 hours and 96 hours post-treatment. At each of these times, whole blood was collected by cardiac puncture and tumours were excised. After collection, 0.2 mL of whole blood was added to a glass scintillation vial to which 1 mL Solvable was added. Samples were incubated for one hour at 55-60°C. 0.1 mL of 0.1 M EDTA-di-sodium salt solution and 0.3 mL to 0.5 mL of 30% hydrogen peroxide in 0.1 mL aliquots were added to the sample. Samples were gently agitated between additions to allow reaction foaming to subside. Samples were incubated for 30 minutes at room temperature. Vials were capped tightly and incubated again at 55 – 60 °C for 1 hour. Samples were cooled to room temperature and 15 mL of ULTIMA Gold added.

For tumour samples, 100 mg of tissue was placed in a 20 mL glass scintillation vial and 2 mL of Solvable added. The samples were incubated at 50 - 60 °C for 1 hour with occasional swirling. Samples were cooled to room temperature and for tumours, 0.3 mL of 30% hydrogen peroxide in three aliquots of 0.1 mL were added. The samples were again incubated at 50 - 60 °C for 1 hour with occasional swirling. Tumours samples were

then treated with 15 mL of ULTIMA Gold. To count radioactivity, a Tri-Carb 2800TR liquid scintillation counter from PerkinElmer was used at the Institute of Medical and Veterinary Science (Adelaide, Australia). All parameters were calculated using the QuantaSmart software package. Where DPM values were missing, the following procedure was performed. Efficiency was calculated by generating an efficiency curve using complete samples (where efficiency = Counts Per Minute/Disintegrations Per Minute), and plotted these values against the provided corresponding transformed spectral index of external standard (tSIE) values.

The pharmacokinetic parameters, C_{max} (maximum plasma concentration), T_{max} (time of maximum plasma concentration), k_{elim} (terminal elimination rate constant), $t_{½}$ (half-life), and AUC (area under the concentration versus time curve) were derived from the blood [^{3}H]-PG545 concentration versus time data using model independent methods. In addition, a linear pharmacokinetic model comprising mono-exponential absorption and bi-exponential disposition and elimination was used to simultaneously model the mean blood concentration versus time data for [^{3}H]-PG545 and the following pharmacokinetic parameters, C_{max} and T_{max}, $t_{½}$, $AUC_{0\text{-}t}$ and $AUC_{0\text{-}\infty}$ for [^{3}H]-PG545 were estimated. Analysis of the tumour [^{3}H]-PG545 concentration versus time data required use of a more physiologically-based pharmacokinetic model such that tumour concentrations were estimated on the basis of the tumour mass being perfused with blood containing [^{3}H]-PG545 and where the blood [^{3}H]-PG545 concentration was changing with time.

LC-MS/MS Assay

Measurement of PG545 in mouse plasma was performed on an API3200 LC-MS/MS (Applied Biosystems) preceded by a Spark Symbiosis Direct On-line Solid Phase Extraction system. Briefly, using a deep-well 96-well plate, 100 µL plasma was mixed with 10 µL standard solution (or water for unknowns) plus 10 µL internal standard solution (PG562 at 100 µg/mL (deionised water for double blank)).This was then mixed for at least 1 min (Ratek Platform Shaker) and incubated at room temperature for 20 mins prior to addition of 25 µL SDS solution (10 mg SDS in 1 mL deionised water) + 75 µL CHAPS solution (50 mg CHAPS in 1 mL deionised water) and 50 µL Proteinase K solution (10 mg Proteinase K in 1 mL 5 mM $CaCl_2$). Following a mixing step this was incubated at 37°C for 2.5 hours prior to addition of 250 µL deionised water and placed into the Spark Symbiosis system. The standard curve range was 0.5 – 100 µg PG545/mL. The T_{max} and C_{max} on each sampling day were determined for each dose level by visual inspection of the mean data. The $AUC_{0\text{-}t}$ was calculated using the trapezoidal method and the plasma concentration $t_{½}$ was estimated based on linear regression of the natural log of the plasma concentration that constituted the terminal phase of the concentration-time profile.
